# A Parameter Communication Optimization Strategy for Distributed Machine Learning in Sensors

**DOI:** 10.3390/s17102172

**Published:** 2017-09-21

**Authors:** Jilin Zhang, Hangdi Tu, Yongjian Ren, Jian Wan, Li Zhou, Mingwei Li, Jue Wang, Lifeng Yu, Chang Zhao, Lei Zhang

**Affiliations:** 1 School of Computer Science and Technology, Hangzhou Dianzi University, Hangzhou 310018, China; jilin.zhang@hdu.edu.cn (J.Z.); 152050103@hdu.edu.cn (H.T.); wanjian@hdu.edu.cn (J.W.); juliy26@hdu.edu.cn (L.Z.); 161050009@hdu.edu.cn (M.L.); 151050064@hdu.edu.cn (C.Z.); 2 Key Laboratory of Complex Systems Modeling and Simulation, Ministry of Education, Hangzhou 310018, China; 3 College of Electrical Engineering, Zhejiang University, Hangzhou 310058, China; 4 School of Information and Electronic engineering, Zhejiang University of Science & Technology, Hangzhou 310023, China; 5 Zhejiang Provincial Engineering Center on Media Data Cloud Processing and Analysis, Hangzhou 310018, China; 6 Supercomputing Center of Computer Network Information Center, Chinese Academy of Sciences, Beijing 100190, China; wangjue@sccas.cn; 7 Hithink RoyalFlush Information Network Co., Ltd., Hangzhou 310023, China; yulifeng@myhexin.com; 8 Financial Information Engineering Technology Research Center of Zhejiang Province, Hangzhou 310023, China; 9 Computer Science Department, Beijing University of Civil Engineering and Architecture, Beijing 100044, China; lei.zhang@bucea.edu.cn

**Keywords:** disturbed machine learning, sensors, dynamic synchronous parallel strategy (DSP), parameter server (PS)

## Abstract

In order to utilize the distributed characteristic of sensors, distributed machine learning has become the mainstream approach, but the different computing capability of sensors and network delays greatly influence the accuracy and the convergence rate of the machine learning model. Our paper describes a reasonable parameter communication optimization strategy to balance the training overhead and the communication overhead. We extend the fault tolerance of iterative-convergent machine learning algorithms and propose the Dynamic Finite Fault Tolerance (DFFT). Based on the DFFT, we implement a parameter communication optimization strategy for distributed machine learning, named Dynamic Synchronous Parallel Strategy (DSP), which uses the performance monitoring model to dynamically adjust the parameter synchronization strategy between worker nodes and the Parameter Server (PS). This strategy makes full use of the computing power of each sensor, ensures the accuracy of the machine learning model, and avoids the situation that the model training is disturbed by any tasks unrelated to the sensors.

## 1. Introduction

Sensor networks have important applications such as environmental monitoring, industrial machine monitoring, body area networks and military target tracking. The design of sensor networks depends on the intended application, so the design of a body area networks must consider energy consumption [1,2], network lifetime [3], hardware, the environment and routing protocols [2]. Tsouri et al. reviewed the major developments in sensor networks and outlined new challenges, surveying the challenges in three diverse categories. The first is the internal platform and underlying operating system. The second is the communication protocol stack. The third is network services, provisioning, and deployment [4]. Machine learning automatically learns programs from data without being explicitly programmed. In the last decade, the use of machine learning has grown from the field of statistics to become a primary mechanism for prediction in diverse applications. In particular, machine learning methods have been widely used in sensors, such as addressing energy-aware communications [5], attack detection and classification [6], task scheduling in wireless sensor networks [7] and generating workloads for cloud computing [8]. Due to the energy and bandwidth constraints of sensors, it is not possible to transmit all the data back to the base station for processing and inference, therefore, it is necessary to apply distributed machine learning in sensors, which can greatly reduce the amount of data in the communication and truly utilize the distributed characteristics of sensors [9]. 

According to the general terms used in distributed machine learning, we also refer to the sensors used for computation as worker nodes in our paper. The main purposes of distributed machine learning in sensors are as follows: (1) solving the problem of insufficient memory of a single worker node to ensure that the data level of terabyte (TB) and above can be processed; (2) using the parallel acceleration training model to drastically decrease the training time. The most important issue is how to achieve parallelization. Data parallelization based on parameter servers (PSs) is a common parallelization scheme in distributed machine learning, whereby the data samples are divided into small datasets and distributed to each worker node, these worker nodes can share the access model parameters. In each iteration of the training, each worker node computes locally and updates its data subset, then submits the local updates to a PS. This PS updates the global model parameters and distributes the new global model parameters to each worker node. In general, the data parallel algorithm is executed in the form of a Bulk Synchronous Parallel (BSP) strategy [10,11]. Worker nodes begin to wait after submitting the local updates, until all worker nodes have submitted the local updates and the PS has updated the global model parameters, then worker nodes will start a new iteration. Thus, BSP leads to load imbalance due to the different performance of worker nodes.

In order to solve the problems existing in BSP, the distributed machine learning asynchronous iterative strategy has been proposed [12,13,14,15], in which worker nodes can start the next iteration by using the local model parameters before receiving the global model parameters. In this strategy, the fault tolerance is increased, which leads to the machine learning model falling into local optima, and the accuracy may not be guaranteed. Ho [16] proposed the Stale Synchronous Parallel (SSP) strategy, which allows each worker node using non-latest global model parameters to reduce the synchronization overhead of submitting the local updates to the PS and strictly controls the number of iterations to ensure the model will converge. Although using the non-latest global model parameters can improve the training speed, the parallel errors are accumulated and the convergence rate is reduced due to the loss of partial updates [17].

Aiming at the problems existing in the abovementioned two strategies, our paper improves SSP and proposes a parameter communication optimization strategy based on the Dynamic Finite Fault Tolerance (DFFT), named Dynamic Synchronous Parallel Strategy (DSP). DSP dynamically adjusts the parameter synchronization strategy between worker nodes and PS based on the performance monitoring model, which effectively reduces the influence of the different performance of worker nodes and ensures the accuracy as well as the convergence rate.

## 2. Related Work 

This section comprehensively introduces the related research in the following four aspects: Section 2.1 describes the distributed machine learning system, Section 2.2 describes the Parameter Server System, Section 2.3 describes SSP, and Section 2.4 describes Robust Optimization.

### 2.1. The Distributed Machine Learning System

Large-scale applications in distributed systems have promoted the development of distributed machine learning. At present, a notable amount of research has been devoted to distributed machine learning systems in both academia and industry.

Chen developed a multi-language machine learning class library called mix-net (MXNet) [18], which is an open source deep learning framework. It can quickly train deep learning models, supports flexible programming models and multiple languages. The MXNet library is portable and lightweight, scalable to multiple Graphics Processing Units (GPUs) and machines. Jia implemented a framework for a deep learning algorithm training method called Caffe [19], which achieves the separation of definition and realization of the model, which greatly simplifies the model training processes [20]. In addition, Caffe supports GPU Compute Unified Device Architecture (CUDA) programming, which further accelerates the model training processes. Ng designed and implemented a multi-GPU clustered distributed deep learning system called Commodity Off-The-Shelf High Performance Computing (COTS HPC) [21] that is based on a Message Passing Interface (MPI). Sparks proposed a novel Machine Learning Interface (MLI) [22], which is designed to accelerate data-centric distributed machine learning. Yin designed a collaborative location-based regularization framework called Colbar [23].

Baidu built a multi-machine GPU training platform called Parallel Asynchronous Distributed Deep Learning (Paddle) [24]. It distributes the data to different machines, coordinates the machine training through PS, and supports data parallelism and model parallelism. Tencent built a deep learning platform called Mariana [25], which includes three frameworks: the deep neural network GPU data parallel framework, the deep convolution neural network GPU data parallel and model parallel framework, and the deep neural network CPU cluster framework. Google developed a large-scale distributed depth learning system called DistBelief [12] to support speech recognition and 2.1 million categories of image classification [26]. On the basis of DistBelief, Google Research implemented and opened up a second-generation large-scale machine learning on heterogeneous distributed systems called TesorFlow [27,28], which has great performance in high-level machine learning calculations with better flexibility and scalability.

In order to ensure consistency, many distributed machine learning systems adopt BSP as the training strategy, which loses the advantages of distributed system performance. A small part of the distributed machine learning systems use completely asynchronous updates as the training strategy, but the model convergence cannot be guaranteed. Thus, a distributed machine learning system for iterative-convergent machine learning algorithms, which is based on the DFFT of machine learning, is still in the initial stage. 

### 2.2. Parameter Server System

**Figure 1:**
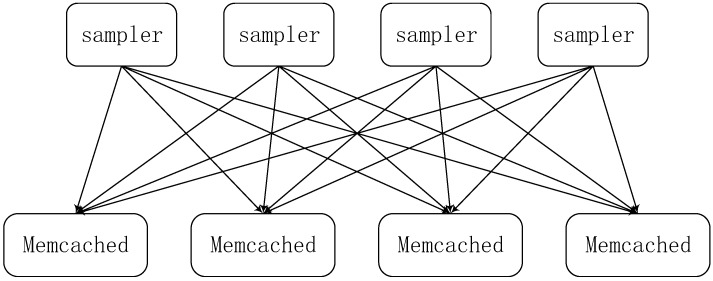
The first-generation parameter server system.

Smola proposed in 2010 a parallel topic model architecture [29], which used the idea of a Parameter Server System, which we call the first-generation parameter server system. The parameter server system is essentially a distributed shared memory system, where each node can access the shared global model parameters through the key value interface, as shown in Figure 1. 

The model uses a distributed Memcached to store the parameters, where each worker node only retains part of the parameters which are required in computing, and they can synchronize global model parameters with each other in this model. However, this parameter server is only a prototype design, the communication overhead is not optimized, and it is not suitable for distributed machine learning.

**Figure 2:**
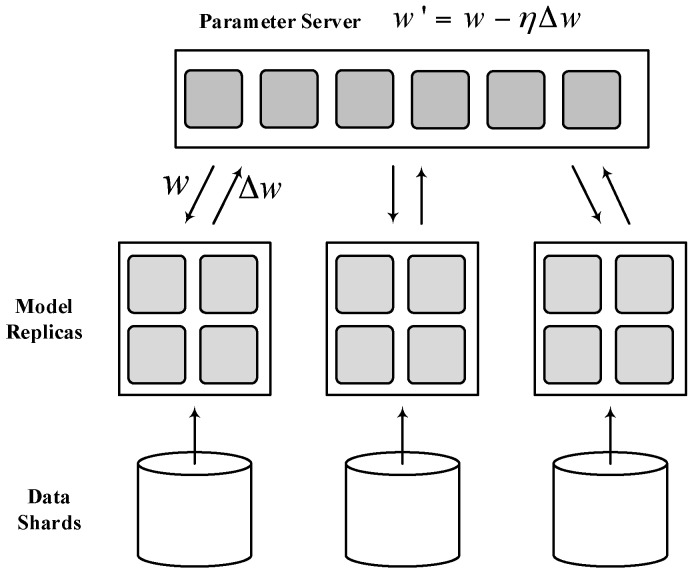
The second-generation parameter server system.

Industry has done a lot of work in improving the Parameter Server System. Dean et al. proposed a second-generation parameter server system in 2012, and developed a deep learning system called DistBelief [12] based on the Parameter Server System. As shown in Figure 2, the system sets up a global parameter server. The deep learning model is distributed stored on worker nodes, the communication between worker nodes is not allowed, and the PS is responsible for the transfer of all parameters. The second-generation parameter server system can solve the problem that the machine learning algorithms are very difficult to be distributed, but because it does not consider the performance differences of each worker node, the utilization of each worker node in the distributed system is still not high.

**Figure 3:**
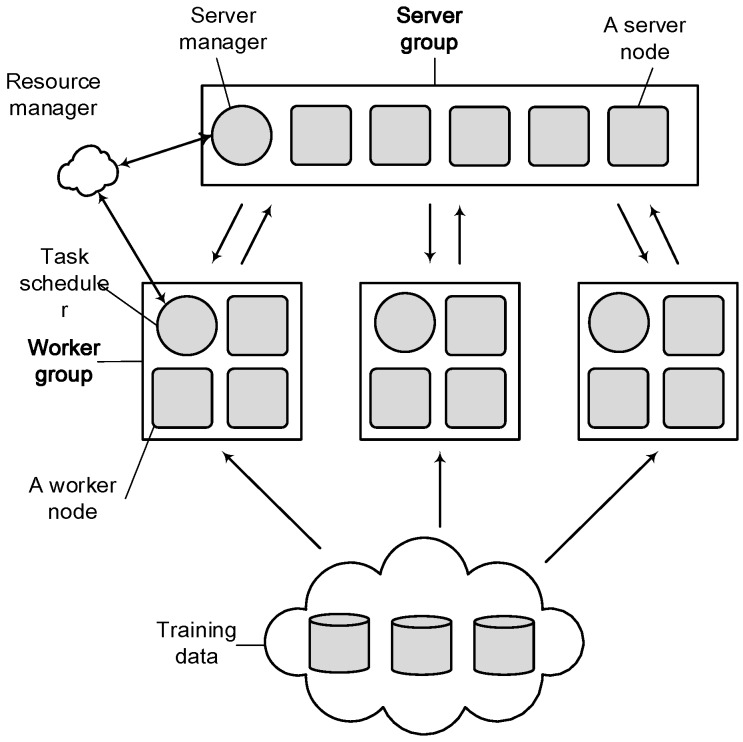
The third-generation parameter server system.

Li proposed a third-generation parameter server system in 2014 [30,31]. As shown in Figure 3, the Parameter Server System provides a more general design, including a parameter server group and multiple worker nodes. 

Among them, each parameter server stores part of the global model parameters, and the server management node manages the entire parameter server group. Multiple worker nodes can run one or more different machine learning algorithms. Like the previous generation of the parameter server system, PS is responsible for the transfer of all parameters. Each worker nodes group sets up a scheduler to assign tasks to worker nodes and monitor the running status. If worker nodes are not responding, the dispatcher performs redistribution of the remaining tasks without re-starting the model training. The Parameter Server System solves the problem of low computing efficiency through scheduling worker nodes, but it can only add or remove the worker nodes. The strategy is too simple and does not consider the situation that the training process of the machine learning model could be disturbed by any unrelated tasks in the cluster.

### 2.3. Stale Synchronous Parallel Strategy

**Figure 4:**
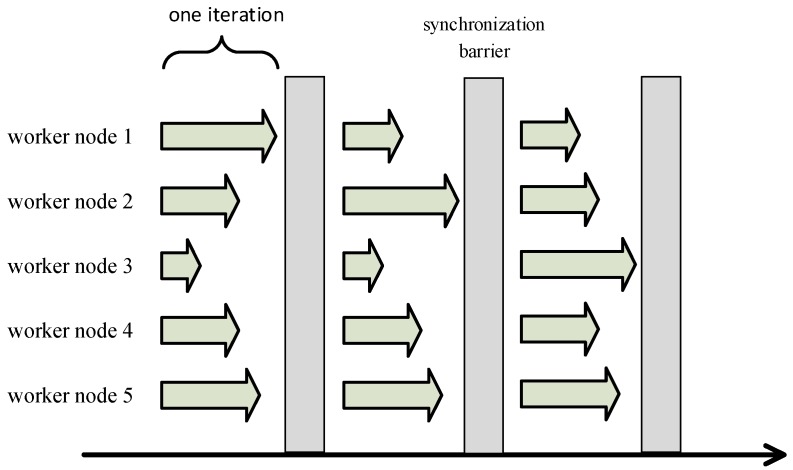
Bulk Synchronous Parallel Strategy (BSP).

The computing power of each worker node may be different in a real environment. For the most commonly used iterative-convergent algorithm in machine learning, the current mainstream distributed machine learning idea is that each worker node trains the model in one iteration and submits the local updates to the PS, then enters the synchronization barrier. When all worker nodes have submitted their local parameters and get the updated global model parameters, the synchronization barrier will be released and the next iteration will start. As shown in Figure 4, the strategy that ensures the global coherence of parameter updates is called the Bulk Synchronous Parallel (BSP) strategy. From the above description we can see that BSP has two obvious defects. The first one is that each iteration requires a lot of communication. Ho [16] has shown that the time required for parameter communication is up to six times the time required for iterative computation in Linear Discriminant Analysis (LDA) theme modeling running on 32 machines. The second one is that all worker nodes that have completed an iteration must wait at the synchronization barrier for the slowest node to finish and then start the next iteration, so the cluster load must be well balanced. However, Chilimbi [14] has shown that even in the load-balanced cluster, some worker nodes will become randomly and unpredictably slower than other worker nodes.

Facing with these disadvantages, Ho proposed SSP [16], which utilizes the fault tolerance of the iterative-convergence algorithm instead of the synchronization barrier for each iteration. Each worker node can directly execute the next iteration after finishing the current iteration. Only when the worker node with the largest number of iterations is faster than the worker node with the least number of iterations s times, all nodes will enter synchronization barrier and be synchronized once, and the s is called the stale threshold. This strategy effectively accelerates the training efficiency of the distributed machine learning model. Wei used this strategy to implement a parameter server system for distributed machine learning [32].

However, the fault tolerance of the iterative-convergence algorithm is finite. SSP does not consider its finiteness. SSP does not limit the number of iterations which may influence the accuracy of the model. Besides, SSP does not consider the dynamic environment, so it cannot cope with external interferences in the training process.

### 2.4. Robust Optimization

The interaction between optimization and machine learning is one of the most important developments in modern computing science [33]. Machine learning is not just a user of optimization technology, but also a producer of new optimization ideas. Sra et al. [33] describes optimization models and algorithms for machine learning such as first-order methods, random approximation, convex relaxation, and also attention to the use of Robust Optimization. 

Robust Optimization is an active and efficient methodology for optimization under uncertainty that has been a challenging area of research in recent years. Robust Optimization will accept a suboptimal solution in order to ensure that the solution is still feasible and nears to the best when the data changes. Bertsimas and Sim [34] proposed a central model for Robust Optimization based on the cardinality constrained uncertainty. Their model ensured certainty and probability, and flexibly adjusted the protection level of probability bounds of constraint violation. Büsing and D’Andreagiovanni [35] generalized and refined the model by Bertsimas and Sim by considering a multi-band uncertainty set, and their Robust Optimization on realistic network design instances performs very well. In recent years, Robust Optimization has also been used in communication networks. Bauschert et al. [36] provided a wide introduction to the topic of network optimization under uncertainty via Robust Optimization.

## 3. Communication Optimization

In this section, we first apply the Stochastic Gradient Descent algorithm (SGD), which is widely used in large-scale machine learning [37], to analyze the feasibility of SSP in theory, and obtain the DFFT of the iterative-convergence algorithm. Then, we introduce the improvement of SSP in detail, and propose a parameter communication optimization strategy, called DSP, based on the DFFT. Finally, we present a distributed machine learning system in sensors using DSP and the idea of parameter server based on Caffe [19].

### 3.1. Theoretical Analysis

Most of the machine learning programs can be transformed to iterative-convergent programs. They can be expressed as follows:

(1) $$L=f(X,M)=f(I_{i=1}^{N}\{x_{i},y_{i}\},M)$$

where *N* is the total number of the dataset, $\left\{x_{i},y_{i}\right\}$ is a sample of the dataset that $y_{i}$ only in the labeled dataset, *M* is the machine learning model.

The scale of the dataset and the model is very large, so the parallel strategy and distributed training are needed. If the stale threshold is set to *s*, the worker node *p* with *t* iterations can access the model ${\tilde{M}}_{p,t}$ which is composed of the initial model and the updates. The model of SSP is as follows:

(2) $${\tilde{M}}_{p,t}=M_{0}+\left[\sum_{i=1}^{t-s-1}\sum_{j=1}^{P}u_{j,i}\right]+\left[\sum_{(i,j)\in U_{p,t}}^{}u_{j,i}\right]$$

$U_{p,t}$ is the subset of the updates submitted by all P worker nodes from iteration *t* − *s* to *t* + *s* − 1. $\left[\sum_{i=1}^{t-s-1}\sum_{j=1}^{P}u_{j,i}\right]$ is the correct updates of the previous iteration, $\left[\sum_{(i,j)\in U_{p,t}}^{}u_{j,i}\right]$ is the best-effort updates of the current iteration.

Next, we analyze the feasibility of SGD in DSP. For a convex function $L=f(M)=\sum_{c=1}^{C}f_{c}(M)$, we use SGD to compute the minimizer $M^{*}$ of the model. The gradient of each worker node is represented by $\nabla f_{c}$, we assume $f_{c}$ is convex, the stale threshold is set to s, and the performance factor of worker nodes is α. The updates of c iterations is $u_{c}:=-\eta_{c}\nabla_{c}f_{c}({\tilde{M}}_{c})$. Then, similar to the derivation in [38,39], we obtain the regret R[*M*] as follows :

(3) $$\begin{array}{l} R[M]:=\left[\frac{1}{C}\sum_{c=1}^{C}f_{c}({\tilde{M}}_{c})\right]-f(M^{*})\leq \sum_{c=1}^{C}\langle \nabla f_{c}({\tilde{M}}_{c}),{\tilde{M}}_{c}-M^{*}\rangle \end{array}$$

Under suitable conditions ($f_{c}$ is *L*-Lipschitz and bounded diameter $D\left(x\|x\prime \right)\leq F^{2}$), the regret of DSP is bounded by:

(4) $$R[M]\leq \sigma L^{2}\sqrt{C}+F^{2}\frac{\sqrt{C}}{\sigma }+2\sigma L^{2}{\left[\left(\frac{s}{\alpha }+1\right)P\right]}^{2}+4\sigma L^{2}\left(\frac{s}{\alpha }+1\right)P\sqrt{C}$$

And the step size is set to $\eta_{c}=\frac{\sigma }{\sqrt{c}}$, where $\sigma =\frac{F}{L\sqrt{2\left(\frac{s}{\alpha }+1\right)P}}$ with constants *F* and *L,* we obtain the DSP theorem as follows:

(5) $$\;R[M]\leq 4FL\sqrt{2\left(\frac{s}{\alpha }+1\right)PC}$$

The method shows that the model trained by DSP can converge to $O(\sqrt{C})$, which means that DSP can ensure the convergence of distributed machine learning. The theorem in [38] only considers the upper limit of the number of error updates, and does not take the DFFT into the consideration, the convergence rate needs to be guaranteed by strict constant adjustment. The proposed method (Equation (5)) improves SSP based on the DFFT, and solves the two problems mentioned in Section 2.3. In our paper, the weak threshold *w* is used to represent the number of iterations performed by the worker node with the worst performance, and the performance factor α is used to represent the difference in the performance of worker nodes. If the performance of each worker node in the cluster is similar, α is close to 1, and the stale threshold *s* cannot be effectively converged, we take the weak threshold *w* instead of the stale threshold *s* as the constraint condition of SSP. If the performance of worker nodes is largely different, then α is large, at this time, we can increase the stale threshold *s* according to Equation (5) which can also ensure a certain convergence rate, then Equation (5) can be amended to:

(6) $$R[M]\leq \left\{ \begin{array}{l} 4FL\sqrt{2\left(\frac{s}{\alpha }+1\right)\mathrm{PC}},\;\alpha \gg 1\\ 4FL\sqrt{2\left(w+1\right)PC},\alpha \approx 1 \end{array}\right.$$

The weak threshold *w* ensures the finite of the fault tolerant, α is calculated from the performance monitoring model and will change from time to time during the training of the distributed machine learning to ensure the dynamics of the fault tolerance.

### 3.2. Dynamic Synchronous Parallel Strategy

#### 3.2.1. Problems of SSP

In Section 2.3, we have briefly introduced the two problems of SSP, here we take a typical distributed machine learning model training as an example to illustrate the problems. 

**Figure 5:**
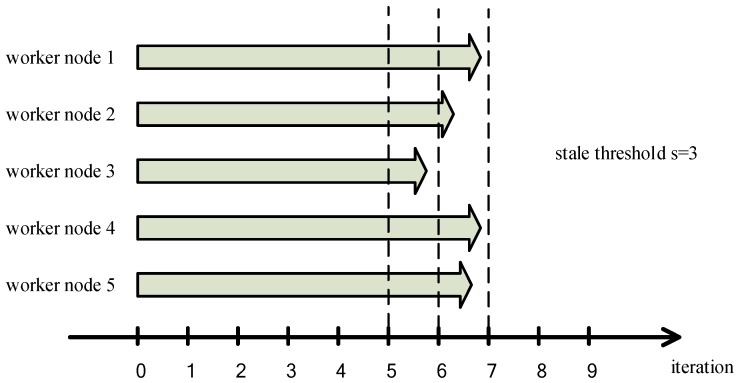
Stale Synchronous Parallel (SSP) is not optimized for the cluster with similar performance worker nodes.

The first problem is that SSP is not optimized for the cluster that is composed of similar performance worker nodes. As shown in Figure 5, there are five worker nodes to train machine learning, the coordinate axis refers to the number of iterations performed by each worker node after the last synchronization barrier. We can clearly see that the performance of each worker node is similar. If the stale threshold is set to 3 or more, each worker node will perform a number of iterations without updating the global model parameters since SSP does not set a single worker node threshold. The fault tolerance of the iterative-convergent algorithm is abused, and the finite of the fault tolerant is neglected. Accuracy of the model is seriously decreased and the convergence of the model is not guaranteed. 

**Figure 6:**
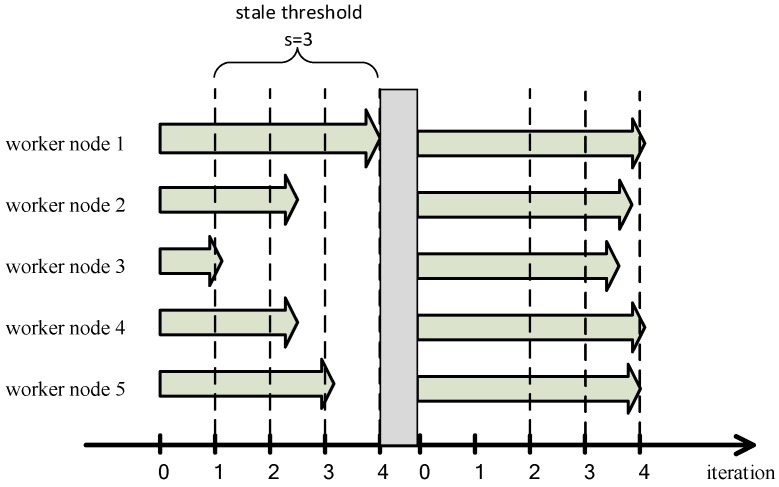
SSP cannot cope with external interference in the process of training model.

The second problem is that SSP cannot cope with external interferences in the training process. As shown in Figure 6, the difference between iterations of the worker node 1 and iterations of the worker node 3 reaches the stale threshold *s*, the worker node 1 waits for the other worker nodes to complete their iterations, then PS performs a global model parameters synchronization, and worker nodes enter the next iteration with the new global model parameters. However, in a new iteration, worker nodes 3 due to some reasons (e.g., the completion of other unrelated computing tasks) improved its computing performance, at this time the number of iterations performed by each worker node is not much different, worker nodes will perform a considerable number of iterations because the stale threshold *s* cannot be reached, which ignores the dynamics of the fault tolerance, and ultimately leads to a serious decrease in the accuracy of model training or even no convergence.

#### 3.2.2. Improvements of SSP

**Figure 7:**
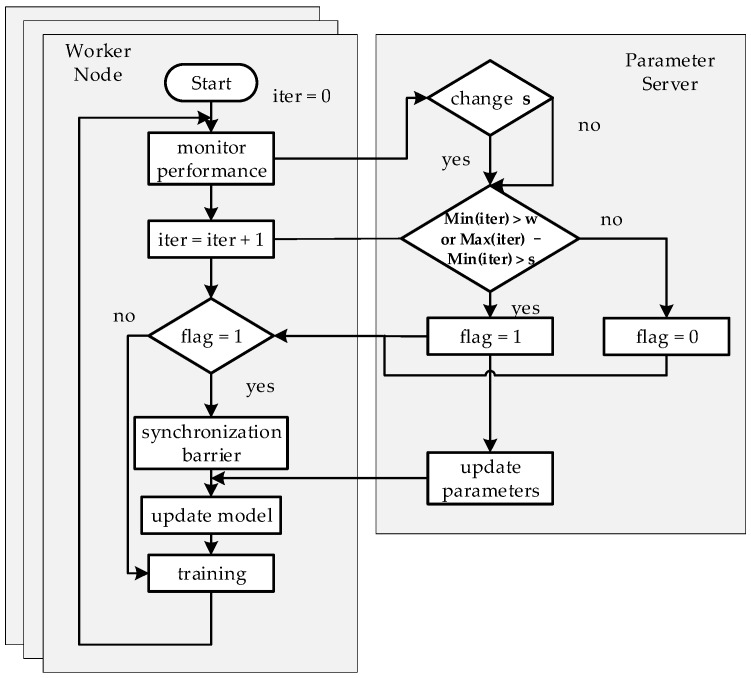
We select a worker node and the PS to show the flow diagram of our optimization strategy procedure.

We propose the Stale Synchronous Parallel Strategy, named DSP, based on the DFFT. This strategy can effectively solve the problems of SSP in distributed machine learning model training. The block diagram of out optimization strategy procedure is shown in Figure 7. Here we describe the solutions in DSP for the two problems mentioned previously.

**Figure 8:**
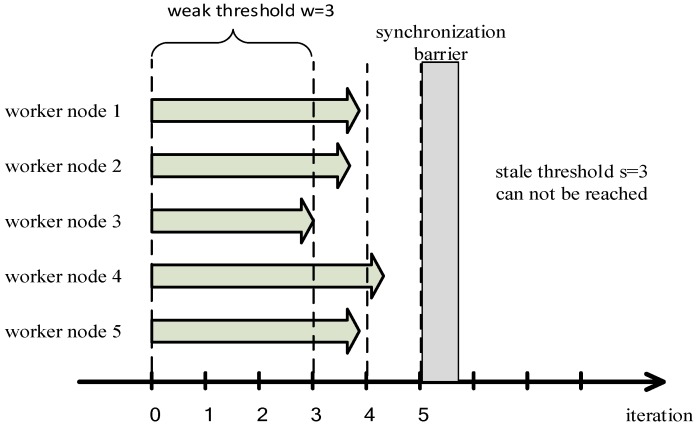
We solve the problem of that using SSP to train distributed machine learning model in the cluster composed with the similar performance worker nodes has the low efficiency.

In order to achieve a finite fault tolerance and solve the low efficiency problem when the cluster is composed of similar performance worker nodes we increase the conditions for entering the synchronization barrier to two: (1) the worker node that trains a smallest number of iterations (which means the worst performance node) completes the iterations w times (we call w the weak threshold); (2) the worker node with the largest number of iterations is faster than the worker node with the smallest number of iterations s times, where *s* is the stale threshold. All worker nodes will enter the synchronization barrier when meeting any of the two conditions, they will update the global model parameters once when they finish their current iteration. As shown in Figure 8, if the computing performance of all worker nodes is similar, the stale threshold *s* is disabled, and the worker node 3 with relatively weak computing performance reaches the weak threshold *w*. Therefore, all worker nodes will still enter the synchronization barrier which avoids any serious decrease of accuracy of the machine learning model.

In order to achieve the fault tolerance dynamics and solve the problem that the stale threshold *s* will be disabled when the computing performance of the worker node has changed in the distributed machine learning training, we implemented a performance monitoring model that calls third-party open source software to monitor the current index, such as CPU, memory, network and I/O, for each worker node and decides if it is necessary to change the stale threshold *s* based on the monitored data.

**Figure 9:**
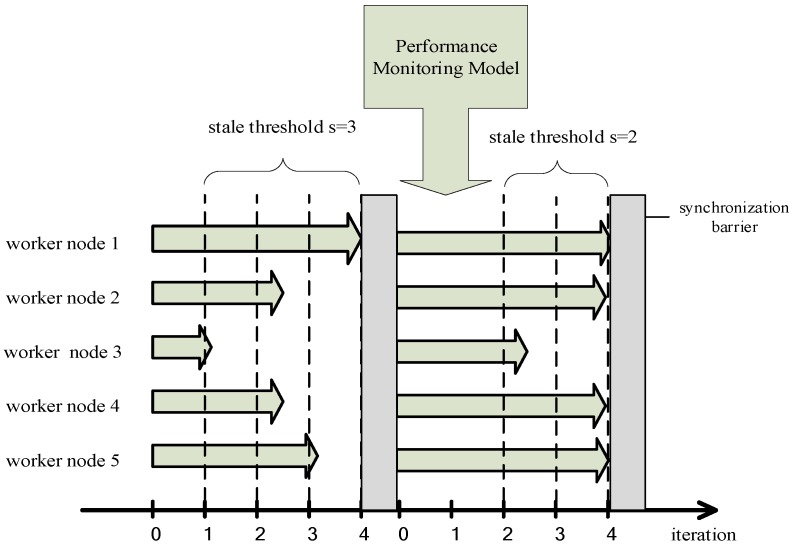
We solve the problem that the stale threshold *s* will be disabled when computing performance of the worker node has changed in the distributed machine learning training.

As shown in Figure 9, when all worker nodes finish a synchronization barrier and start a new iteration, the performance model finds the change in the index of the worker node 3, and estimates that the computing performance of the worker node 3 is increased and the performance difference of each worker node is reduced, so the performance model reduces the stale threshold *s* to 2 to avoid the excessive iteration situation and solves the problem of the stale threshold failure.

### 3.3. Distributed Machine Learning System in Sensors Based on Caffe

**Figure 10:**
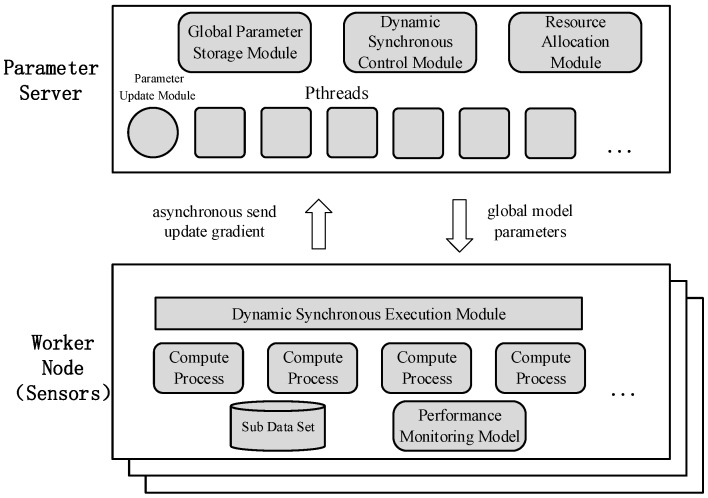
A distributed machine learning system based on Caffe and supports Dynamic Synchronous Parallel Strategy (DSP).

Caffe is a deep learning framework made with expression, speed, and modularity in mind [13]. It was developed by Berkeley AI Research (BAIR) and by community contributors. However, the open source version of Caffe does not support distributed machine learning. We use the idea of PS to implement a distributed machine learning system in sensors based on Caffe that supports DSP. The architecture is shown in Figure 10.

On the PS, the Global Parameter Storage Module is used to store the latest global model parameters. The Dynamic Synchronous Control Module is used to perform DSP to dynamically adjust the stale threshold *s* and the weak threshold *w* of sensors according to the computing performance of each sensor. The Resource Allocation Module is used to analyze the computing performance of each sensor and implement the adjustment strategy of the stale threshold *s* and the weak threshold *w*. The Parameter Update Module is used to compute the global model parameters when all sensors enter the synchronization barrier, where an idle queue is implemented to store the state of the worker node. Each computation process will have a corresponding thread (created by POSIX threads) on PS, which is responsible for communication with the sensor, such as receiving the computing performance of the sensor, the number of iterations per compute process, and so on.

On the sensors, the Sub Dataset is divided from the dataset according to the number of sensors. The Performance Monitoring Model is used to monitor the current index of sensors such as CPU, memory, network, I/O and other performance indicators. The Compute Processes are used to train the machine learning model of data parallelism. The Dynamic Synchronous Execution Module determines whether the number of iterations of the sensor reaches the implementation condition of the synchronization barrier based on the stale threshold *s* and the weak threshold *w* computed by PS. If it is not reached, the sensor will update the model according to the update gradient computed by itself and perform the next iteration. If the threshold is reached, all compute processes will wait after finishing the current iteration until received the latest global model parameters from PS. 

## 4. Experiments and Results

### 4.1. Experimental Environment

The experiments in our paper use the distributed machine learning system introduced in Section 3.3, which is deployed in a cluster of three nodes to simulate sensor nodes. The nodes are connected by Gigabit Ethernet, the operating system of the cluster is CentOS 7.0, and the configuration of the cluster node is 16 AMD (Processor 6136) with a main frequency of 2.4 GHz and 32 GB of memory.

We use the MNIST handwritten digital font dataset [40] as our dataset. The training set contains 60,000 images and the test set contains 10,000 images. The training model is based on the classical LeNet-5 [40], which includes an input layer, an output layer, three convolutional layers, two pool layers, and a full connected layer. The batch size of the training model is configured to 64 and the maximum number of iterations is set to 10,000.

### 4.2. The Finite of the Fault Tolerance

**Figure 11:**
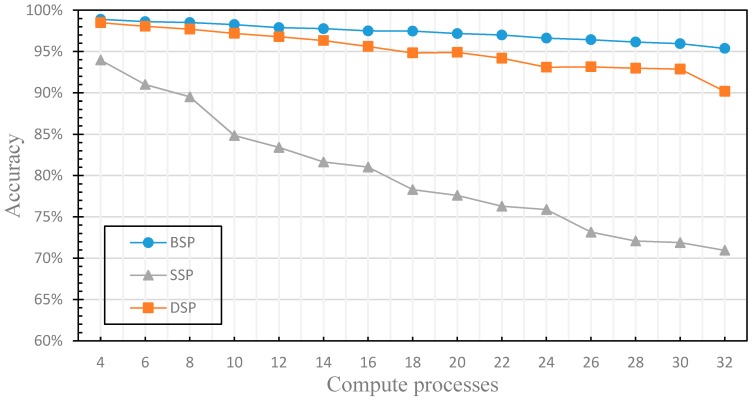
This figure compares the accuracy of the model for machine learning with each parameter communication optimization strategy.

**Figure 12:**
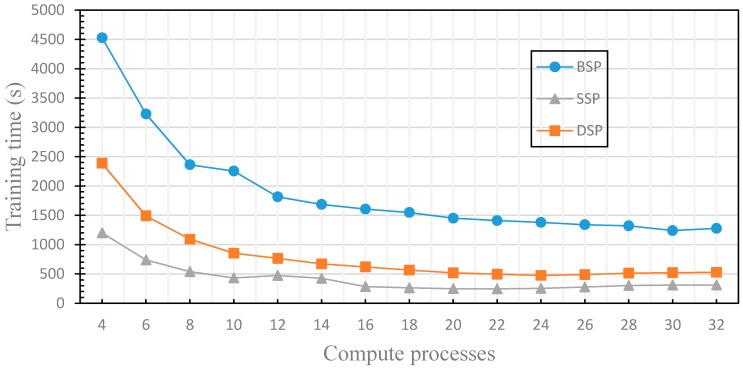
This figure compares the training time of machine learning model training with each parameter communication optimization strategy.

In this section, we compare the performance differences of distributed machine learning with three different parameter communication optimization strategies (including BSP, SSP and DSP) to verify the finite nature of the fault tolerance. We use the three nodes in the cluster to train the distributed machine learning model, where one node acts as a parameter server and two nodes act as worker nodes. By comparing the training time and accuracy of the machine learning model under the different communication optimization strategies, we verify the limitation of the fault tolerance of the iterative learning convergence algorithm, and evaluate the performance of DSP. The experimental results are shown in Figure 11 and Figure 12, where the stale threshold *s* of SSP and DSP is set to 3, the weak threshold *w* of DSP is set to 1.

Figure 11 compares the accuracy of the machine learning model when applying different parameter communication optimization strategies. As shown in the figure, regardless of which parameter communication optimization strategy is used for distributed machine learning model training, the accuracy decreases as the computation processes increase. Figure 12 compares the training time of machine learning model training with each parameter communication optimization strategy. Similarly, regardless of which parameter communication optimization strategy is used, the training time decreases with the increase of computation processes. With the gradual increase of computation processes, the number of communications between worker nodes and PS will be increased. Therefore, when the number of computation processes reaches a certain value, the communication cost is greater than the computing cost, and the training time is no longer reduced. BSP is required to enter the synchronization barrier after each iteration to ensure strong consistency and accuracy, but it also takes a lot of time. SSP leverages the fault tolerance, and the training time is lower, but it is not aware of the finiteness of the fault tolerance, and does not set the weak threshold w, which leads to the low accuracy as the increasing of compute processes. DSP in this experiment does not use the performance monitoring module and cannot dynamically adjust the delay threshold *s*, but it uses the finiteness of the fault tolerance, and sets the weak threshold w, so there is still a good accuracy guaranteed with the increasing of compute processes, the training time is lower than that of BSP and close to that of SSP. In this section, we have experimentally evaluated the finiteness of the fault tolerance of the machine learning iteration-convergence algorithm, solved the problem that SSP has the low efficiency for the distributed machine learning algorithm in clusters that is composed of nodes with similar performance.

### 4.3. The Dynamics of the Fault Tolerance

**Figure 13:**
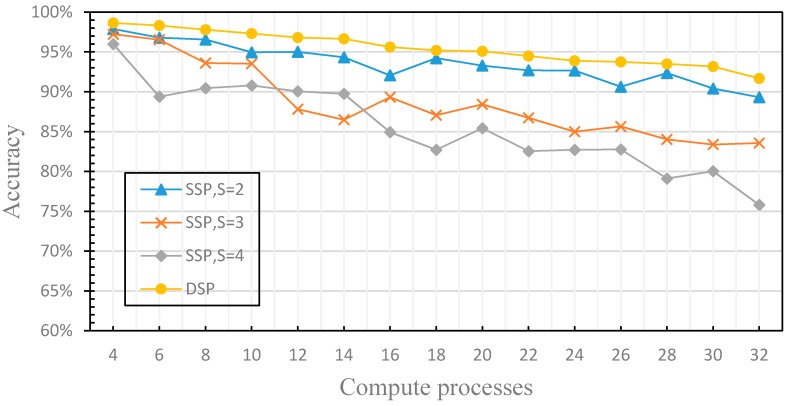
This figure compares the effects of SSP with different stale thresholds and DSP on the accuracy of the distributed machine learning model.

**Figure 14:**
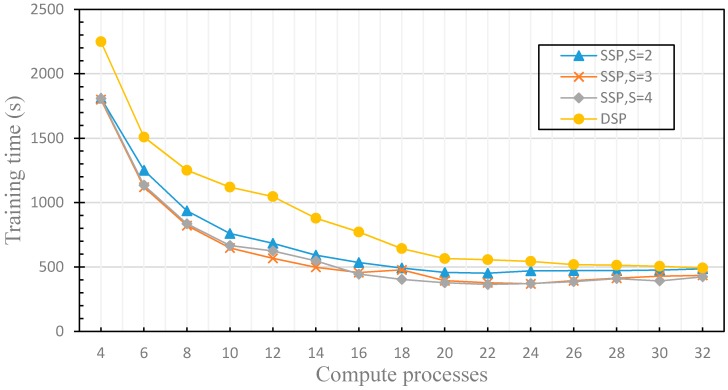
This figure compares the difference of training time between the distributed machine learning model using SSP with different stale thresholds and DSP.

In this section, we compare the performance differences of distributed machine learning with SSP (with the different stale threshold) and DSP to verify the dynamics of the fault tolerance. For SSP, the stale threshold *s* is set to 2, 3 and 4, respectively. For DSP, the stale threshold *s* is dynamically adjusted according to the performance of each worker node, and the weak threshold *w* is set to 1. The experimental results are shown in Figure 13 and Figure 14.

Figure 13 shows the comparison of the effects of SSP with different stale thresholds and DSP on the accuracy of the distributed machine learning model. It can be seen from Figure 13 that the accuracy of the distributed machine learning model using SSP decreases with the increasing number of computation processes, but it fluctuates. This is due to the fact that the stale threshold *s* set by SSP cannot be increased or decreased when the performance of the computing node is changed, according to the analysis of Section 3.2.1.

Hence, when the performance of worker nodes tends to be similar, there will be too many training iterations as s is too large and the training results may fall into a local optimal solution, and the accuracy decreases rapidly. When the difference in worker nodes’ performance increases, the performance of each computing node cannot be fully utilized since s is too small. With the increase of s, it will be more and more difficult to deal with the similar performance of worker nodes, which leads to the decrease of the accuracy. The distributed machine learning model using DSP guarantees the dynamics of the fault tolerance, and uses the performance monitoring model which evaluates the performance difference factors of each worker node to dynamically adjust the stale threshold *s*. Therefore, regardless of whether the number of computation processes increases, the accuracy of distributed machine learning model is maintained. Figure 14 compares the difference of training time between the distributed machine learning model using SSP with different stale thresholds and DSP. The data shows that the training time of the distributed machine learning model using SSP is faster than that of the distributed machine learning model using DSP. This is because SSP ignores the finiteness of the fault tolerance and discards the accuracy to speed up the training time. In order to ensure the accuracy of the model, the distributed machine learning model using DSP sets the weak threshold *w*, which increases the synchronization overhead. Therefore, the training time is longer than that of the distributed machine learning model using SSP, but the training time difference is very small. Using the DFFT, the distributed machine learning model using DSP achieves both high accuracy and satisfactory training time. In this section, we have evaluated the dynamics of the fault tolerance by experiments, solved the problem that SSP has low efficiency when the computing performance of the worker nodes changes dynamically.

## 5. Conclusions

Although the distributed machine learning model training with SSP can improve the utilization of computing nodes and the computing efficiency by reducing communication and synchronization overhead, the cumulative errors can significantly influence the performance of the algorithm and lead to low convergence speed. Frequent communication can reduce the stale threshold and parallel error, thereby improve the performance of the algorithm, but this is subject to the network transmission rate limit.

In this paper, we optimize SSP, extend the fault tolerance of the machine learning iteration-convergence algorithm, propose the DFFT, and implement DSP which is a parameter communication optimization strategy based on the DFFT. DSP dynamically adjusts the stale threshold according to the performance of each worker node to ensure the balance between the computational efficiency and the convergence rate. The weak threshold is added to solve the problem that SSP is inefficient to train the distributed machine learning model in clusters which are composed of nodes with similar performance. Finally, the experimental results show that the efficiency and the convergence rate of DSP are better than that of BSP and SSP under the premise that the accuracy is guaranteed. At present, the convergence rate and accuracy of the distributed machine learning system based on DSP are not very good for large-scale instances. In our future work, we will improve the scalability of this strategy, especially in large-scale sensors systems.

## References

[1] D’Andreagiovanni F., Nardin A. (2015). Towards the fast and robust optimal design of wireless body area networks. Appl. Soft Comput..

[2] Tsouri G.R., Prieto A., Argade N. (2012). On Increasing Network Lifetime in Body Area Networks Using Global Routing with Energy Consumption Balancing. Sensors.

[3] Natalizio E., Loscri V., Viterbo E. (2008). Optimal placement of wireless nodes for maximizing path lifetime. IEEE Commun. Lett..

[4] Yick J., Mukherjee B., Ghosal D. (2008). Wireless sensor network survey. Comput. Netw..

[5] Pandana C., Liu K.R. (2005). Near-optimal reinforcement learning framework for energy-aware sensor communications. IEEE J. Sel. Areas Commun..

[6] Junejo K.N., Goh J. Behaviour-Based Attack Detection and Classification in Cyber Physical Systems Using Machine Learning. Proceedings of the ACM International Workshop on Cyber-Physical System Security.

[7] Van Norden W., de Jong J., Bolderheij F., Rothkrantz L. (2005). Intelligent Task Scheduling in Sensor Networks. Proceedings of the 2005 8th International Conference on Information Fusion.

[8] Yin J., Lu X., Zhao X., Chen H., Liu X. (2015). BURSE: A bursty and self-similar workload generator for cloud computing. IEEE Trans. Parall Distrib. Syst..

[9] Di M., Joo E.M. (2007). A Survey of Machine Learning in Wireless Sensor Netoworks from Networking and Application Perspectives. Proceedings of the 2007 6th International Conference on Information, Communications & Signal Processing.

[10] McColl W.F. (1995). Bulk synchronous parallel computing. Abstract Machine Models for Highly Parallel Computers.

[11] Gerbessiotis A.V., Valiant L.G. (1994). Direct bulk-synchronous parallel algorithms. J. Parallel Distrib. Commun..

[12] Dean J., Corrado G., Monga R., Chen K., Devin M., Mao M., Senior A., Tucker P., Yang K., Le Q.V. (2012). Large Scale Distributed Deep Networks. Advances in Neural Information Processing Systems.

[13] Ahmed A., Aly M., Gonzalez J., Narayanamurthy S., Smola A. Scalable Inference in Latent Variable Models. Proceedings of the International conference on Web search and data mining (WSDM).

[14] Chilimbi T., Suzue Y., Apacible J., Kalyanaraman K. Project Adam: Building an Efficient and Scalable Deep Learning Training System. Proceedings of the Usenix Conference on Operating Systems Design and Implementation.

[15] Cui H., Tumanov A., Wei J., Xu L., Dai W., Haber-Kucharsky J., Ho Q., Ganger G.R., Gibbons P.B., Gibson G.A. (2014). Exploiting Iterative-Ness for Parallel ML Computations. Proceedings of the ACM Symposium on Cloud Computing.

[16] Ho Q., Cipar J., Cui H., Lee S., Kim J.K., Gibbons P.B., Gibson G.A., Ganger G., Xing E.P. (2013). More Effective Distributed ML via a Stale Synchronous Parallel Parameter Server.

[17] Xing E.P., Ho Q., Xie P., Wei D. (2016). Strategies and principles of distributed machine learning on big data. Engineering.

[18] Chen T., Li M., Li Y., Lin M., Wang N., Wang M., Xiao T., Xu B., Zhang C., Zhang Z. (2015). Mxnet: A flexible and efficient machine learning library for heterogeneous distributed systems. arXiv.

[19] Jia Y., Shelhamer E., Donahue J., Karayev S., Long J., Girshick R., Guadarrama S., Darrell T. (2014). Caffe: Convolutional Architecture for Fast Feature Embedding. Proceedings of the 22nd ACM international conference on Multimedia.

[20] Szegedy C., Liu W., Jia Y., Sermanet P., Reed S., Anguelov D., Erhan D., Vanhoucke V., Rabinovich A. Going Deeper with Convolutions. Proceedings of the IEEE Conference on Computer Vision and Pattern Recognition.

[21] Coates A., Huval B., Wang T., Wu D.J., Ng A.Y., Catanzaro B. Deep Learning with COTS HPC Systems. Proceedings of the IEEE Conference on Computer Vision and Pattern Recognition.

[22] Sparks E.R., Talwalkar A., Smith V., Kottalam J., Pan X., Gonzalez J., Franklin M.J., Jordan M.I., Kraska T. MLI: An API for Distributed Machine Learning. Proceedings of the 2013 IEEE 13th International Conference on Data Mining (ICDM).

[23] Yin J., Lo W., Deng S., Li Y., Wu Z., Xiong N. (2014). Colbar: A collaborative location-based regularization framework for QoS prediction. Inf. Sci..

[24] Yu K. Large-Scale Deep Learning at Baidu. Proceedings of the ACM International Conference on Information & Knowledge Management.

[25] Zou Y., Jin X., Li Y., Guo Z., Wang E., Xiao B. (2014). Mariana: Tencent deep learning platform and its applications. Proc. VLDB Endow..

[26] Le Q.V. (2013). Building High-Level Features Using Large Scale Unsupervised Learning. Proceedings of the 2013 IEEE International Conference on Acoustics, Speech and Signal Processing (ICASSP).

[27] Abadi M., Agarwal A., Barham P., Brevdo E., Chen Z., Citro C., Corrado G.S., Davis A., Dean J., Devin M. (2016). Tensorflow: Large-scale machine learning on heterogeneous distributed systems. arXiv.

[28] Abadi M., Barham P., Chen J., Chen Z., Davis A., Dean J., Devin M., Ghemawat S., Irving G., Isard M. TensorFlow: A System for Large-Scale Machine Learning. Proceedings of the 12th USENIX Symposium on Operating Systems Design and Implementation (OSDI).

[29] Smola A., Narayanamurthy S. (2010). An architecture for parallel topic models. Proc. VLDB Endow..

[30] Li M., Andersen D.G., Park J.W., Smola A.J., Ahmed A., Josifovski V., Long J., Shekita E.J., Su B.Y. Scaling Distributed Machine Learning with the Parameter Server. Proceedings of the Usenix Conference on Operating Systems Design and Implementation.

[31] Li M., Andersen D.G., Smola A., Yu K. Communication Efficient Distributed Machine Learning with the Parameter Server. Proceedings of the International Conference on Neural Information Processing Systems.

[32] Wei J., Dai W., Qiao A., Ho Q., Cui H., Ganger G.R., Gibbons P.B., Gibson G.A., Xing E.P. (2015). Managed Communication and Consistency for Fast Data-Parallel Iterative Analytics. Proceedings of the Proceedings of the Sixth ACM Symposium on Cloud Computing.

[33] Sra S., Nowozin S., Wright S.J. (2012). Optimization for Machine Learning.

[34] Bertsimas D., Sim M. (2004). The price of robustness. Oper. Res..

[35] Büsing C., D’Andreagiovanni F. (2012). New Results about Multi-Band Uncertainty in Robust Optimization. Experimental Algorithms, Proceedings of the 11th International Symposium, SEA 2012, Bordeaux, France, 7–9 June 2012.

[36] Bauschert T., Busing C., D’Andreagiovanni F., Koster A.C., Kutschka M., Steglich U. (2014). Network planning under demand uncertainty with robust optimization. IEEE Commun. Mag..

[37] Gemulla R., Nijkamp E., Haas P.J., Sismanis Y. (2011). Large-Scale Matrix Factorization with Distributed Stochastic Gradient Descent. Proceedings of the 17th ACM SIGKDD international conference on Knowledge Discovery and Data Mining.

[38] Dai W., Kumar A., Wei J., Ho Q., Gibson G., Xing E.P. High-Performance Distributed ML at Scale through Parameter Server Consistency Models. Proceedings of the Twenty-Ninth AAAI Conference on Artificial Intelligence.

[39] Li M., Zhou L., Yang Z., Li A., Xia F., Andersen D.G., Smola A. Parameter Server for Distributed Machine Learning. Proceedings of the Big Learning NIPS Workshop.

[40] Cun Y.L., Boser B., Denker J.S., Howard R.E., Habbard W., Jackel L.D., Henderson D. (1990). Handwritten Digit Recognition with a Back-Propagation Network. Advances in Neural Information Processing Systems.

